# Genetic Elements at the Alpha-Synuclein Locus

**DOI:** 10.3389/fnins.2022.889802

**Published:** 2022-07-11

**Authors:** Jordan Prahl, Gerhard A. Coetzee

**Affiliations:** Department of Neurodegenerative Disease, Van Andel Institute, Grand Rapids, MI, United States

**Keywords:** Parkinson’s disease, GWAS, alpha-synuclein (*SNCA*), rs356182, neurodevelopment

## Abstract

Genome-wide association studies have consistently shown that the alpha-synuclein locus is significantly associated with Parkinson’s disease. The mechanism by which this locus modulates the disease pathology and etiology remains largely under-investigated. This is due to the assumption that *SNCA* is the only driver of the functional aspects of several single nucleotide polymorphism (SNP) risk-signals at this locus. Recent evidence has shown that the risk associated with the top GWAS-identified variant within this locus is independent of *SNCA* expression, calling into question the validity of assigning function to the nearest gene, *SNCA*. In this review, we examine additional genes and risk variants present at the *SNCA* locus and how they may contribute to Parkinson’s disease. Using the *SNCA* locus as an example, we hope to demonstrate that deeper and detailed functional validations are required for high impact disease-linked variants.

## Introduction

Since the earliest genome-wide association studies (GWAS), the alpha-synuclein (*SNCA*) locus on chromosome 4 has consistently stood out as a risk-associated region for Parkinson’s disease (PD) (Nalls et al., 2014, 2019). Although ethnic variations manifest in PD clinical features and demographic-specific differences in variant associations, the *SNCA* locus as a whole is largely conserved across racial backgrounds (Nalls et al., 2019; Ben-Joseph et al., 2020; Foo et al., 2020). While most GWAS meta-analyses primarily measured the risk estimates in Caucasians (Nalls et al., 2019), a study of the genetic risk factors between Asian and Caucasian individuals showed significant overlap (Foo et al., 2020). We therefore concentrated here on data from Caucasians. We do, however, realize that there is a substantial gap in our mechanistic and risk appreciation of inter-ethnic variation in general, and specifically at the SNCA locus with respect to PD risk (Sirugo et al., 2019). The presence of alpha-synuclein protein (α-SYN) in pathological Lewy bodies, coupled with *SNCA*-associated familial forms of PD, has led to the foregone conclusion that the risk for sporadic PD at this locus is driven by a *SNCA*-associated mechanisms (Polymeropoulos et al., 1997; Spillantini et al., 1997; Grenn et al., 2020). This predictable conclusion fails to account for the often-complex biology of GWAS-identified risk variants. These variants are mostly assigned an identifier based on the nearest gene on linear DNA, but rarely is the risk mechanism for the variant ever confirmed through further investigation. Further, these single nucleotide polymorphisms (SNPs) occur most often in non-coding regions of the genome and within dynamically active genetic enhancers (Pierce et al., 2018). Approximately two thirds of genetic enhancers skip the nearest gene entirely and interact exclusively with distal promotors (Gusev et al., 2016; Zhu et al., 2016), and often interact with multiple promotors depending on the specific-context (Chepelev et al., 2012; Ernst, 2012). An example of a prominent risk variant which lacked a confirmed mechanism is the PD risk-SNP rs356182 at the *SNCA* locus. We have recently demonstrated through the use of the dopaminergic neuron cell model, LUHMES, that the risk associated with rs356182 is largely independent of *SNCA* expression, contrary to assumptions (Prahl et al., 2021). In this review, we hope to demonstrate that making oversimplified assumptions about SNPs interacting exclusively with the nearest genes may lead to incomplete understanding about the true risk mechanism.

## Parkinson’s Disease

It has been over 200 years since the “shaking palsy” was first described by James Parkinson, but his namesake disease continues to challenge researchers (Parkinson, 1817). PD was initially described as a motor disease due to its characteristic motor symptoms. Over the years, researchers have gained a deeper understanding of PD and reclassified it as a complex neurodegenerative disease (Goetz, 2011). Protein aggregates made primarily of fibrillated α-SYN were discovered in the dopaminergic neurons of the substantia nigra pars compacta of PD patients (Spillantini et al., 1997). These aggregates, known as Lewy bodies, are now considered the molecular hallmark of the disease in addition to degeneration of the dopaminergic neurons (Lewandowsky, 1912; Spillantini et al., 1997). Despite improved understanding of this disease, there remains no practical way to stop or reverse it, leaving physicians with only palliative treatment options (Rizek et al., 2016; Armstrong and Okun, 2020; Iarkov et al., 2020).

Twin studies have attributed about 27% of PD risk to genetics (Goldman et al., 2019). This risk can be separated into two categories; sporadic PD and familial PD. Familial PD, accounting for ∼10% of all PD cases, can be traced to mutations in one, or a few, key genes, and about 10% of all PD cases have a known family history following these specific mutations that are either dominant or recessive (Thomas and Beal, 2007; Spatola and Wider, 2014). Studying gene-disease interactions in the familial context gave researchers great insight into the pathophysiology of PD. The first identified monogenic, autosomal dominant, form of PD is caused by mutations and copy number variations (CNVs) of *SNCA* (Polymeropoulos et al., 1997).

The etiology of sporadic PD remains largely elusive. Comprehensive GWASs have been linking SNPs with PD to demystify the ‘missing’ heritability of sporadic PD (Zhang et al., 2018; Nalls et al., 2019; Sirugo et al., 2019; Foo et al., 2020; Ohnmacht et al., 2020). Each SNP contributes only minimally to the relative risk of the disease; therefore, it is likely a complex relationship between all the SNPs and risk factors that determines one’s true propensity for PD (Nalls et al., 2019). However, some SNPs may have more robust impacts on disease risk, progression, or phenotype than other SNPs. After years of analysis and meta-analysis, one locus has consistently been identified as the most significant association with PD: namely the *SNCA* locus and specifically the SNP rs356182 (Nalls et al., 2019; Foo et al., 2020).

## The *SNCA* Locus

Recent GWAS have revealed 90 independent risk signals in 78 different loci for PD throughout the human genome (Nalls et al., 2019). The most tantalizing locus identified by these GWAS is the *SNCA* locus for several reasons. First, as previously mentioned, the *SNCA* protein product is found in great abundance in Lewy bodies, which are the molecular hallmark of PD (Spillantini et al., 1997). Second, mutations and CNVs of *SNCA*, although rare, are one of the most well-known causes of familial PD (Polymeropoulos et al., 1997). Finally, the meta-analysis of 17 PD-GWAS indicated that the rs356182 SNP at the *SNCA* locus has the most significant correlations to PD and nearly the highest magnitude risk (Nalls et al., 2019). In addition to rs356182, many other SNPs at this locus have been identified to be independently relevant to PD.

### Single Nucleotide Polymorphisms

Within the *SNCA* locus, there are numerous SNPs associated with PD, but due to the linkage disequilibrium of this locus, only a few independent risk signals have been identified. Pihlstrom et al. (2018) suggests there are three independent risk signals, rs356182, rs763443, and rs2870004, which we describe in greater detail below (Pihlstrom et al., 2018). In addition to these “independent” risk signals, conditional SNP analysis has suggested that rs7681154 is another independent variant of interest (Nalls et al., 2014; Soldner et al., 2016). Soldner et al. (2016) analyzed the genotype-dependent binding affinity of transcription factors (TF) at the *SNCA* locus and reported that the intronic SNP rs356168 had the most robust effect on TF binding and was therefore highly likely to modulate expression of nearby genes (Soldner et al., 2016). In this section, we summarize the current depth of knowledge regarding these five SNPs. These SNPs do not represent an exhaustive list of the SNPs which may be worth investigating within this locus; they are merely examples of variants which have been pulled from the literature as potentially relevant, but which have had strikingly little research into their mechanism. For each SNP, we describe the GWAS data, a summary of the published literature, and eQTL data from the GTEx Portal.^1^ A summary of the eQTL results can be found in Supplementary Table 2. Only *SNCA* and *MMRN1* are summarized in Supplementary Table 2 because those are the genes with data for these SNPs on the GTEx Portal, and we focus here on the substantia nigra data since that is the most immediately relevant to PD.

#### rs356182

**FIGURE 1:**
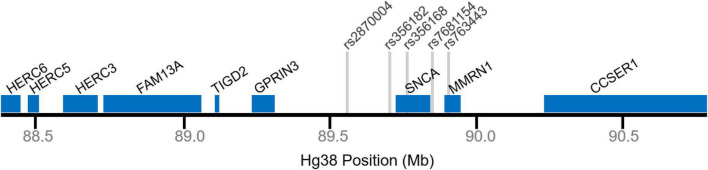
Genomic map of the *SNCA* locus. Selected gene positions displayed in blue. Gray lines denote relative single nucleotide polymorphism (SNP) positions.

The most significant risk-SNP identified for PD is rs356182 (meta-p-value = 1.85 × 10^–82^) (Nalls et al., 2019). It is an A > G polymorphism on chromosome 4, positioned in the intergenic region centromeric of *SNCA* (Figure 1). The minor allele frequency for the G-allele (which is also the reference- and the risk-allele) is approximately 36% in Caucasians, and the odds ratio is 1.34 (95% CI: 1.30–1.38) (Cheng et al., 2016; Nalls et al., 2019). Although it is not the only significant or independent SNP in the locus, computational analysis suggests rs356182 is independently functional (Pihlstrom et al., 2018). According to the eQTL data from the GTEx Portal, rs356182 does not significantly correlate (*P* < 0.05) to *SNCA* expression in most brain regions including the substantia nigra. In the brain regions that do show a significant correlation (Spinal cord c-1 and cerebellar hemisphere) *SNCA* is more highly expressed in the presence of the protective A-allele. As previously mentioned, published comprehensive analysis of this SNP and its mechanism are currently lacking and primarily focused on its relationship to *SNCA* expression (Supplementary Table 1). Alternatively, GTEx data suggests that rs356182 is significantly correlated to *MMRN1* with the risk G-allele promoting expression in nearly every tested brain region including the substantia nigra (Supplementary Table 2).

ChIP-seq for histone 3 lysine 27 acetylation (H3K27ac), an epigenetic marker commonly used to identify genetic regulatory elements, shows a peak at rs356182 which indicates the presence of a genetic enhancer (Pierce et al., 2018). This potential enhancer was corroborated by Pihlstrom et al. (2018) when they published a similar H3K27ac peak overlapping with a neuronal-specific ATAC-seq peak. There appears to be a significant genotype-phenotype relationship, with the risk genotype GG correlating strongly with a tremor-dominant endophenotype, while AA and AG were more heavily weighted toward the postural and gate instability phenotype (Cooper et al., 2017). The same study also showed the GG genotype was associated with a slower progressing motor phenotype and lowered *SNCA* gene expression. These results may seem paradoxical in that the “risk-allele” (G) is associated with higher incidences of PD but more favorable phenotypes and outcomes of the disease (Cooper et al., 2017). Contradictory studies, however, have stated that rs356168 is a more potent indicative marker for *SNCA* expression than rs356182, which may indicate that rs356182 acts in mechanisms independent or in addition to *SNCA* modulation (Soldner et al., 2016).

#### rs356168

One of the other top ranked SNPs in PD-GWAS is the SNP, rs356168, which falls within an enhancer in intron 4 of the *SNCA* gene (Figure 1). This SNP is a G > A variant in which the minor A-allele is the protective allele (OR = 0.79: 0.76–0.81, *p* = 2.7 × 10^–50^) (Nalls et al., 2019). Studies of alcohol dependencies showed a robust correlation between rs356168 and dopamine response in certain regions of the brain (Wilcox et al., 2013). Particularly, the A-allele was correlated with a more robust brain response in the alcohol taste response test and ultimately a more severe illness (Wilcox et al., 2013).

Conflicting results have been published regarding rs356168 and its control of the expression of *SNCA*. Soldner et al. (2016) found that the protective A-allele was inhibiting the expression of SNCA through interactions with the transcription factors EMX2 and NKX6-1. They also reported that conditioning the other SNPs to rs356168 had a robust effect on those SNPs achieving significance. In this analysis, rs356182 still reached significance, but rs7681154 was the condition top hit. However, Glenn et al. (2017) attempted to validate Soldner’s findings and instead reported the opposite effect on *SNCA* expression. In their analysis of post-mortem cortex tissue, they report that the protective A-allele was correlated with an increase in *SNCA* expression (Glenn et al., 2017). This conclusion mirrors the finding from rs356182 in which the protective allele there also correlates with higher SNCA expression (Cooper et al., 2017). The GTEx Portal reports that rs356168 did not achieve a significant correlation to *SNCA* expression in the substantia nigra but did significantly correlate to *MMRN1* expression (Supplementary Table 2).

#### rs7681154

rs7681154 is an A > C polymorphism with a MAF for the C-allele ∼38%, located in the promotor region of the *SNCA* gene (Figure 1). Beyond that, not much is known about rs7681154. The latest GWAS data from Nalls et al. (2019) reported the unconditioned OR for rs7681154 to be 1.0 with a meta-*p*-value of 0.854 (Nalls et al., 2019). Conditional analysis of rs7681154 upon the top SNP in the locus (rs356182) demonstrated that it was conditionally independent (Nalls et al., 2014). Likewise, Soldner et al. (2016) reported rs7681154 to maintain independent significance when conditioned on rs356168. According to the eQTL data available from GTEx Portal, rs7681154 does not have a significant correlation to *SNCA* or *MMRN1* expression in any of the tested brain regions (Supplementary Table 2). Despite being confirmed as an independent risk-SNP for PD, there are currently no published attempts at identifying a risk mechanism for rs7681154 (Supplementary Table 1).

#### rs763443

rs763443 is one of the SNPs Pihlstrom et al. (2018) identified as an independent risk signal at the *SNCA* locus (Pihlstrom et al., 2018). It is a C > T variant with a MAF at 45%, located in an intron of *MMRN1* (Figure 1). This SNP failed to reach significance in association to PD in the Nalls et al. meta-analysis (Nalls et al., 2019). The only other notable reference to this SNP is an analysis of disease heterogeneity based on genotype of the risk-signals identified by Pihlstrom et al. (2018). They found that this SNP failed to explain any of the disease heterogeneity amongst a large cohort of newly diagnosed PD patients (Szwedo et al., 2020). According to GTEx, rs763443 does not significantly correlate to *SNCA* or MMRN1 expression in the substantia nigra (Supplementary Table 2).

#### rs2870004

rs2870004 is another SNP identified as an independent risk signal by Pihlstrom et al. (2018). It is an A > T variant with a MAF of 23%, located in the intergenic space between *SNCA* and *GPRIN3*. Again, rs2870004 failed to reach significance in the Nalls et al. (2019) meta-analysis. Like rs763443, the only other publication which references this SNP is the analysis of disease heterogeneity, and again they found that this SNP failed to explain any of the disease heterogeneity (Szwedo et al., 2020). GTEx data suggests that rs2870004 is not significantly correlated to *SNCA* or *MMRN1* expression within the substantia nigra (Supplementary Table 2).

### Genes

In addition to the SNPs at the *SNCA* locus, other genes are nearby which may be regulated, either instead of, or in addition to, *SNCA* by nearby enhancers (Figure 1). The *SNCA* locus triplication cohort of patients includes triplication of 12 other genes [5 protein-coding (Olgiati et al., 2015)] and the partial disruption of both the *HERC6* gene and the *CCSER1* gene (Zafar et al., 2018). *SNCA*-replication cohorts are rare and vary in replication size, but even in the most stringent multiplication cohorts, *MMRN1* is also included in the duplicated regions (Ross et al., 2008). At the very least, this may cause inflation of the effect attributed to *SNCA* regarding PD in these families. In this section we will explore some of the other prominent genes which occur within this locus. These genes do not represent a comprehensive list of all of the known genetic elements in this region but serve as an example of potential alternative risk-associated targets at this locus.

#### SNCA

Alpha synuclein is by far the most studied gene in this locus (Supplementary Table 1). In fact, there are several other reviews which do a phenomenal job of relaying the current state of *SNCA* research (Goedert, 2001; Fernagut and Chesselet, 2004; Cheng et al., 2011; Dehay et al., 2016; Emamzadeh, 2016; Oliveira et al., 2021), therefore we will not go into exhaustive detail here. The following represents only a broad overview of *SNCA* and its product, α-SYN.

As stated above, *SNCA* encodes the protein, α-SYN, which is a small 140 amino acid protein. Under normal conditions, α-SYN is expressed in mature dopaminergic neurons and participates in a variety of biological functions. Most notably, α-SYN is found in synaptic terminals where it functions in synaptic vesicle trafficking (Bellani et al., 2010; Burre et al., 2010; Nemani et al., 2010; Wu et al., 2014). α-SYN has also been observed in the nucleus of cells, where it has transcription factor-like activity and DNA repair functionality (Siddiqui et al., 2012; Kim et al., 2014; Pinho et al., 2019). It has been described as a general regulator of gene expression for genes associated with dopamine synthesis (Baptista et al., 2003; Surguchev and Surguchov, 2017). Another notable function of α-SYN is its role in regulating neuronal cell proliferation (Lee et al., 2003; Rodriguez-Losada et al., 2020; Prahl et al., 2022). Many of these mechanisms indicate chaperone-like activity for α-SYN, necessary for neuronal survival (Kanaan and Manfredsson, 2012).

Under pathological conditions, α-SYN becomes misfolded and aggregates into Lewy bodies, which demonstrates prion-like propagation. Mutations and replications of the *SNCA* gene lead to monogenic familial PD (Conway et al., 1998; Olgiati et al., 2015; Zafar et al., 2018). Recent evidence suggests that Lewy bodies sequester α-SYN from the nucleus to the cytoplasm, preventing it from functioning to repair double-stranded DNA breaks, resulting in eventual apoptosis (Schaser et al., 2019). This contributes to a growing body of evidence that the synuclein pathology in PD is a loss-of-function (LOF) mechanism (Benskey et al., 2016). Mice with double knockout of the SNCA gene experience very early gastrointestinal dysfunction indicating a role in the gut (Kuo et al., 2010). Whatever the mechanism, α-SYN is undoubtably a participant in the pathogenesis of PD. The proposed influence of SNCA to PD has veiled the potential for alternative/additional mechanisms involving other genes in this locus. It has even been suggested that α-SYN is merely a “bystander” in sporadic PD (Riederer et al., 2019).

#### FAM13A

*FAM13A* (Family with Sequence Similarity 13 Member A) is a protein coding gene on chromosome 4 centromeric to *SNCA*. The FAM13A protein is expressed in many human tissues and cell types, but most notable to this review is its expression in excitatory neurons according to GTEx. *FAM13A* has not previously been associated with PD (Supplementary Table 1), but it’s activity in the substantia nigra and within neurons makes it a candidate risk-gene. *FAM13A* functions by regulating GTPase-mediated signal transduction (Amin et al., 2016).

#### MMRN1

Multimerin 1 (*MMRN1*) is a protein coding gene adjacent to *SNCA*. As the name would imply, *MMRN1* primarily functions in multimers of the protein in platelets and the endothelium of blood vessels. Not surprisingly, *MMRN1* is primarily associated with blood disorders. According to GTEX, *MMRN1* is almost non-existent in the substantia nigra. There is, however, some expression in the blood brain barrier providing a potential avenue for which this gene may modulate PD. *MMRN1* is also replicated in the *SNCA*-CNV families, showing a 6-fold increase in mRNA expression compared to controls (Ross et al., 2008; Olgiati et al., 2015; Zafar et al., 2018). The triplication seems to result in adverse expression of *MMRN1* when it shouldn’t be present, potentially contributing to the PD pathology seen in this cohort.

#### HERC

The HERC family of human proteins can be separated into two groups based on structure and function: the large HERCs (1 and 2) and the small HERCs (3–6). The *HERC3, 5*, and *6* genes are located near *SNCA* and entangled with the *SNCA*-triplication cohort (Figure 1). These genes are all expressed ubiquitously, with particularly high expression of *HERC2* in the brain and *HERC6* in the testis, and their proteins localize to cytoplasmic puncta (Hochrainer et al., 2005, 2008). It is believed that the HERC proteins all interact together in late endosomes and lysosomes (Hochrainer et al., 2008). *HERC3* and *HERC5* appear to have a role in inflammatory and immune responses (Sanchez-Tena et al., 2016). *HERC2*, although not at this locus, has a known role in PD as it interacts with *LRRK2* (Imai et al., 2015).

#### CCSER1

*CCSER1* (Coiled Coil Serine Rich protein 1) has very little expression in the substantia nigra or brain in general according to GTEX. Deletion of *CCSER1* results in mitotic cell division defects, and likewise overexpression elicits cellular division (Patel et al., 2013; Santoliquido et al., 2021). Other disease associations include diabetic neuropathy, substance abuse, peripheral artery disease, and cancer. There are few publications which acknowledge CCSER1 (Supplementary Table 1) and fewer still that relate to PD. Of those that do discuss CCSER1 in the context of PD, they are primarily focused on its disruption in the triplication cohort (Zafar et al., 2018; Suzuki et al., 2020).

#### GPRIN3

At the *SNCA* locus, *GPRIN3* (G-protein-regulated inducer of neurite outgrowth 3) is the next gene centromeric to rs356182. It is a mediator of DRD2 function and striatal neurons lacking *GPRIN3* show increased dendritic arborization and decreased neuronal excitability (Karadurmus et al., 2019). *GPRIN3* has a CTCF binding site in its non-coding region whose H3K27ac is sensitive to rotenone exposure (Freeman and Wang, 2020). *GPRIN3* is within the triplicated region in several of the PD *SNCA*-triplication cohorts (Ibanez et al., 2009; Gwinn et al., 2011; Olgiati et al., 2015). According to GTEX, GPRIN3 has robust expression in the cerebellum but minimal expression in the substantia nigra.

#### TIGD2

*TIGD2* (Tigger transposable element derived 2) belongs to the Tigger subfamily of the Pogo superfamily of human DNA-mediated transposons. The exact function of this gene is not known but transposons can participate in “changing the cells identity” (Bourque et al., 2018). It seems possible that *TIGD2* may interact in cell differentiation and in that way would fit into the model of PD being a developmental disorder (Schwamborn, 2018; von Linstow et al., 2020). At present, *TIGD2* is primarily associated with breast cancer (Xiao et al., 2018; Nakamura et al., 2019). According to GTEX, *TIGD2* has some expression in the cerebellum but very little expression in the substantia nigra.

## The *SNCA* Assumption

The assumed mechanism regarding risk associated with rs356182 posits that rs356182 most likely regulate *SNCA* expression and that’s the only reason it is associated with PD. As stated above, due to the proximity of rs356182 to the known PD-associated gene *SNCA*, it is easy to assume that the risk arises from this interaction. One could even argue that the identification of the enhancer containing rs356182 strengthens this assumption. The PD GWAS locus browser by Grenn et al. provides the following insight:

*“SNCA is the most likely candidate in this region. SNCA missense mutations and multiplications cause Parkinson’s disease. Additionally, SNCA is found inside Lewy bodies which is one of the pathological hallmarks of Parkinson’s disease. Several studies have already investigated the potential effect of the SNCA common variants and the increased expression of SNCA*” (Grenn et al., 2020).

This insight is entirely focused on *SNCA* and completely disregards the potential for alterative mechanisms. In fact, the lack of in-depth functional characterization of rs356182 is likely a result of this assumption, which fails to address several counter points.

The first topic to address in response to the *SNCA* assumption is enhancer dynamics. Being an intergenic SNP, rs356182 most likely confers risk through modulation of transcription factor binding within an enhancer. We, and others, have demonstrated that an enhancer exists in this locus just as predicted (Pierce et al., 2018; Pihlstrom et al., 2018). The issue that arises is in the assignment of enhancer interactions to the nearest gene, in this case *SNCA*. Enhancers do not exclusively interact with the most proximal gene (Brynedal et al., 2017; Liu et al., 2019; Bhattacharya and Love, 2020). In fact, enhancers often skip the nearest gene all together, or interact with multiple gene promotors at once. Likewise, a single gene promotor may interact with multiple enhancers (Chepelev et al., 2012; Ernst, 2012). To assume that the distal enhancer at the *SNCA* locus is exclusively regulating the *SNCA* gene is an oversimplification of what is likely a much more complex mechanism. Additionally, enhancer activity is highly variable and completely context dependent; determined by cell type, cell cycle, timing through differentiation, and external factors (Pierce et al., 2020). This explains why, until recently, no enhancer was ever observed at this locus. Previous investigation of the H3K27ac may have looked in the wrong cell type or at the wrong time in the cells and completely missed the enhancer.

Another topic to consider is the differentiation process. As we know, differentiation is a seismic event in the lifecycle of a cell. Gene expression and enhancer activity change rapidly, the effects of which may be immediately apparent or may not manifest until years later. Genes critical to neuronal differentiation are reportedly downregulated in PD (Verma et al., 2020). Recent theories have linked PD (an age-related disorder) to events during differentiation during embryogenesis (Schwamborn, 2018). Human neuron population densities are known to vary by a large degree, and it has been suggested that these differences in neuron populations are established during neurogenesis *in utero* (von Linstow et al., 2020). This theory provides the perfect platform to address rs356182 activity, timing, and disease association. We propose that rs356182 (and its encompassing enhancer) participate in neuronal differentiation, establishing the neuronal density in the substantia nigra, and set up individuals for risk of PD after subsequent “second hits” later in life. The hypothesis being, those born with a smaller population of dopaminergic neurons are more susceptible to developing disruptions in dopamine signaling following natural neuronal cell loss due to age or external stimuli than those born with a larger population (von Linstow et al., 2020; Prahl et al., 2021).

## Conclusion

While effort has certainly been dedicated to investigating particular elements of the *SNCA* locus, there remain glaring vacancies in the current field of research. In addition to numerous other protein coding genes and SNPs in this region, there are enhancers which are known to have highly dynamic activity. We hope to compel future researchers to consider the alternative hypothesis, that SNCA is not the only important element in this locus. In that pursuit, we recommend functional characterization of the PD-associated risk SNPs at this locus with consideration of cell type and timing in mind (Pierce et al., 2020). Soldner et al. (2016) provide an excellent example of how to interrogate specific SNPs of interest by identifying potential transcription factors likely to bind at the locus and determining how those interactions are affected by genotype (Soldner et al., 2016). As we discussed, assuming SNPs modulate expression of only the most proximal gene is an oversimplification, so a more comprehensive technique (e.g., RNAseq) is recommended to determine to true scope of genotype effect. Additionally, the expression-genotype relationship data is currently lacking a full catalog of tissues, and more effort must be placed on identifying the gene targets of the risk-associated SNPs within the proper cell types. With a better understanding of the complete pathological mechanisms surrounding GWAS hits, we can better inform future therapeutic targets for PD.

## Author Contributions

This review was developed in collaboration between both authors. JP wrote the manuscript with supervision of GC. JP and GC edited the manuscript over several rounds of discussion.

## Conflict of Interest

The authors declare that the research was conducted in the absence of any commercial or financial relationships that could be construed as a potential conflict of interest.

## Publisher’s Note

All claims expressed in this article are solely those of the authors and do not necessarily represent those of their affiliated organizations, or those of the publisher, the editors and the reviewers. Any product that may be evaluated in this article, or claim that may be made by its manufacturer, is not guaranteed or endorsed by the publisher.

## Footnotes

1
https://www.gtexportal.org/home/snp/[SNP]
