# Supplementary material for: Genetic Elements at the Alpha-Synuclein Locus
Source: Front Neurosci. 2022 Jul 11;16:889802. doi: 10.3389/fnins.2022.889802 (PMC9309432; doi:10.3389/fnins.2022.889802)
Supplement: Supplementary file 1 [file Data_Sheet_1.docx]

**Supplemental Tables**

**Table S1. Genetic elements at the *SNCA* locus.** Included in the table is the elements’ identity, genomic position, and the number of search results on PubMed. The PubMed search was updated on May 20, 2022. The search term for each item was “IDENTITY”[Title/Abstract] and then the more stringent "Parkinson's"[Title/Abstract] AND " IDENTITY "[Title/Abstract] where IDENTITY corresponds to the item in the IDENTITY column below.

| Element | Identity | Position (GRCh38) | PUBMED Results (IDENTITY ONLY) | results (identity + parkinson’S) |
| --- | --- | --- | --- | --- |
| GENE | SNCA | 89724099 - 89838304 | 1,291 | 891 |
| GENE | FAM13A | 88725960 - 89057185 | 115 | 0 |
| GENE | HERC5 | 88457119 - 88506163 | 99 | 1 |
| GENE | MMRN1 | 89879511 - 89954614 | 39 | 4 |
| GENE | HERC6 | 88378852 - 88443097 | 33 | 1 |
| GENE | HERC3 | 88523843 - 88708539 | 24 | 0 |
| GENE | CCSER1 | 90127394 - 91605295 | 20 | 1 |
| SNP | rs356182 | 89704960 | 8 | 6 |
| GENE | GPRIN3 | 89236383 - 89307800 | 5 | 2 |
| SNP | rs356168 | 89753280 | 3 | 2 |
| GENE | TIGD2 | 89111169 - 89114901 | 2 | 0 |
| SNP | rs2870004 | 89550094 | 2 | 1 |
| SNP | rs763443 | 89898810 | 1 | 1 |
| SNP | rs7681154 | 89842552 | 0 | 0 |

**Table S2. GTEx Portal eQTL associations between SNPs and the nearby genes, *SNCA* and MMRN1.** The value in each cell is the given P-value for each correlation within the substantia nigra brain tissue. P-values less than 0.05 are highlighted in green.

| SNP | SNCA | MMRN1 |
| --- | --- | --- |
| rs356182 | 0.298 | 0.007 |
| rs356168 | 0.500 | 0.020 |
| rs2870004 | 0.090 | 0.909 |
| rs763443 | 0.067 | 0.054 |
| rs7681154 | 0.900 | 0.200 |
